# Evolutionary mode for the functional preservation of fast-evolving *Drosophila* telomere capping proteins

**DOI:** 10.1098/rsob.210261

**Published:** 2021-11-17

**Authors:** Balázs Vedelek, Ákos Kovács, Imre M. Boros

**Affiliations:** ^1^ Department of Biochemistry and Molecular Biology, University of Szeged, Szeged, Hungary; ^2^ Institute of Biochemistry, Biological Research Centre, Szeged, Hungary

**Keywords:** Terminin, fast evolution, Ob-fold, Ver, Moi, DTL

## Abstract

DNA end protection is fundamental for the long-term preservation of the genome. In vertebrates the Shelterin protein complex protects telomeric DNA ends, thereby contributing to the maintenance of genome integrity. In the *Drosophila* genus, this function is thought to be performed by the Terminin complex, an assembly of fast-evolving subunits. Considering that DNA end protection is fundamental for successful genome replication, the accelerated evolution of Terminin subunits is counterintuitive, as conservation is supposed to maintain the assembly and concerted function of the interacting partners. This problem extends over *Drosophila* telomere biology and provides insight into the evolution of protein assemblies. In order to learn more about the mechanistic details of this phenomenon we have investigated the intra- and interspecies assemblies of Verrocchio and Modigliani, two Terminin subunits using *in vitro* assays. Based on our results and on homology-based three-dimensional models for Ver and Moi, we conclude that both proteins contain Ob-fold and contribute to the ssDNA binding of the Terminin complex. We propose that the preservation of Ver function is achieved by conservation of specific amino acids responsible for folding or localized in interacting surfaces. We also provide here the first evidence on Moi DNA binding.

## Graphical abstract

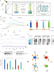

## 1. Introduction

The increasing number of sequenced species has greatly accelerated progress in the field of protein evolution studies. The accumulated knowledge has caused a shift in the earlier held concept that ancient proteins are more conserved, while younger ones are characterized by signs of accelerated evolution. According to the presently held view, in addition to phylogenetic age, several other factors, such as expression level, protein importance and connectivity, also affect the speed of evolution [1–6] (summarized in [7]). Despite these interesting findings, it is still generally accepted that most of the proteins that participate in fundamental cellular functions, such as transcription, translation or replication, are ancient and well conserved [1]. Conservation suggests strong selection acting on these sequences; one could say that a minor change in the sequence is enough to compromise the function of these multi-protein machineries.

Telomere maintenance has a particularly fascinating relation to replication and would be expected to be conserved. Nonetheless, different variations of telomere maintenance have evolved [8]. A common element of these is that repeats of sequences form the specific telomeric DNA and proteins bind to these sequences. The participating proteins form a DNA protecting cap and contribute to the elongation of telomeric DNA. Besides preventing DNA loss due to activities of exonucleases from chromosome ends, the protective cap also prevents chromosome fusions. The ends of linear chromosomes resemble DNA double-stranded breaks; therefore, without telomere capping the DNA repair mechanisms would recognize and fuse those, causing genomic instability [9]. Proteins are also needed to elongate the telomeric DNA in order to solve the ‘end replication problem’ which otherwise would cause progressive telomere shortening with successive cycles of DNA replication. The ‘end replication problem’ arises from the inability of the replication machinery to completely synthetize the lagging strand after the removal of the last RNA primer at a chromosome end [10,11]. Most eukaryotes use a specific reverse transcriptase called telomerase to elongate DNA at chromosome ends. Telomerase uses its own RNA as template repeatedly to synthesize tandem repeats of a short GC rich sequence, constituting telomeric DNA [12,13]. Due to the activities of telomerase and the replication machinery, telomeric DNA consists of double-stranded (ds) and single-stranded (ss) regions. In most organisms with telomerase, proteins of the Shelterin complex bind to repeats of the telomeric DNA, forming a protective cap on telomeres and regulating telomeric DNA elongation [9]. However, besides telomerase and Shelterin, other means of telomere elongation and protection are also known [8,14,15]; *Drosophila*, for example, have lost telomerase during evolution and solve the end replication problem by integration of three telomeric retrotransposons at chromosome ends. These ‘domesticated’ transposons, HeT-A, TART and TAHRE form arrays of repeats at telomeric regions [16,17]. As a consequence, in the absence of short telomeric repeats, in flies sequence-specific DNA binding of Shelterin-like proteins cannot provide telomere protection. Instead, *Drosophila* species use a functionally analogous but evolutionarily new complex called Terminin, which binds to DNA in a sequence-independent manner to provide the protective cap required for preventing chromosome fusions. As such, in *Drosophila* Terminin fulfils the same role as Shelterin in other organisms [18].

The Terminin complex is hypothesized to consist of four fast-evolving and one conserved subunit, which are HOAP [19], HipHop [20], Verrocchio (Ver) [21], DTL [22]/Modigliani (Moi) [23] and HP1 [24], respectively. From these proteins, two stable subcomplexes can be assembled *in vitro* (HOAP–HipHop–HP1 and Ver–Moi), but not a full Terminin complex [25]. Zhang *et al.* [26] recently described another protein, Tea, which interacts with Moi and Ver, forming the Moi–Tea–Ver (MTV) complex. MTV is believed to contribute to telomere maintenance and Tea was found to be essential for the DNA binding of MTV. In parallel Cicconi *et al.* [27] showed that a Ver homodimer is able to bind ssDNA, while Moi has no ssDNA binding activity. It remains an open question whether MTV and HOAP–HipHop–HP1 are two distinct complexes or are both subcomplexes of a Terminin holocomplex. Nevertheless, as these assemblies are linked by the shared function of telomere capping and maintenance, we consider each of these proteins as components of the Terminin complex. Although both Terminin subcomplexes bind to DNA in a sequence-independent manner, they differ in their affinity towards different DNA structures: HOAP–HipHop–HP1 binds to dsDNA, while MTV binds ssDNA [20,26,27].

Terminin proteins fulfil the same function of telomere capping as Shelterin, however, Terminin subunits evolve significantly faster than Shelterin proteins do (electronic supplementary material, figure S1). This contradiction was noted and Saint-Leandre *et al.* [28] suggested that the fast evolution of the HOAP protein was caused by the adaptive evolution required to silence telomeric transposons. While this might explain the driving force behind the paradox, the question of how the function of the fast-evolving proteins is preserved remains unanswered. We sought a solution for this puzzle by identifying conserved regions of a Terminin subunit Ver, and by analysing interactions between selected components of Terminin and DNA. Based on our results we suggest that Terminin proteins preserve their functions because despite their overall fast evolution, they maintain conserved structure and surfaces required for interactions.

## 2. Results

### 2.1. Both intra- and interspecies Ver–Moi subcomplexes of Terminin show preferential binding to ssDNA

**Figure 1.**
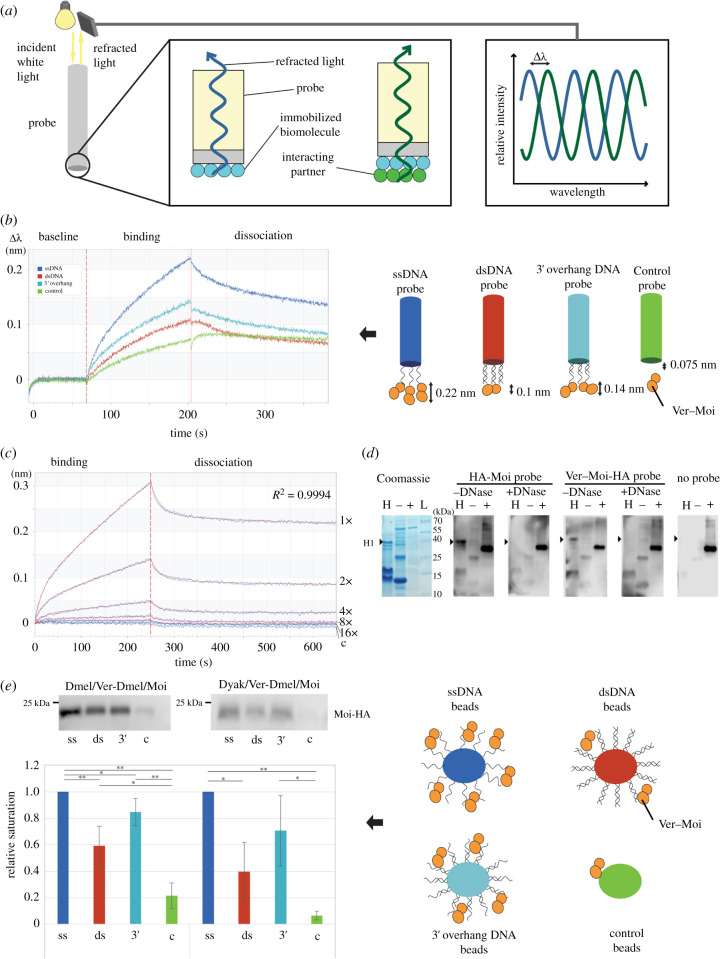
Both *D. melanogaster* Ver–Moi dimer and *D. yakuba* Ver–*D. melanogaster* Moi hybrid dimer binds ssDNA with higher affinity than dsDNA. (*a*) Schematic illustration of Biolayer interferometry. Biomolecules bound to the surface of a probe cause a phase shift in the refracted light captured by the detector. From the interference of refracted light waves, the degree of the phase shift, reflecting the density of the bound biomolecules can be determined. (*b*) Biolayer interferometry results show that Ver–Moi heterodimer binds to ssDNA with higher affinity than dsDNA. Dark blue (ssDNA) curve shows larger response compared to the red (dsDNA) or light blue (dsDNA with 3′ ss overhang), indicating the highest amount of Ver–Moi heterodimer binding to ssDNA. (*c*) Binding curves indicate two DNA binding surfaces of Ver–Moi complex on ssDNA. Recordings were made using 1×, 2×, 4×, 8× and 16× diluted Ver–Moi dimer samples (blue curve). The obtained data fits well with the heterogenic ligand model (*R*^2^ = 0.9994) (red curve). Using the model two *K*_d_ values could be calculated (*K*_d1_ = 1.29 × 10^−7^ M; *K*_d2_ = 1.57 × 10^−6^ M), suggesting that Ver–Moi dimer has at least two DNA binding surfaces. (*d*) Result of the Far western experiment indicates indirect interaction of Moi with H1 histone via DNA. HA-Moi and Ver–Moi-HA interact with H1 histone when DNA is present, but nuclease treatment diminishes the interaction. L: molecular weight marker; H: *Drosophila* histone extract; −: negative control, bacterial cell extract with lysozyme; +: positive control cell extract with HA-HipHop). (*e*) DNA affinity pull-down experiments show preferential binding of Ver–Moi dimer to ssDNA. Representative western blots and statistical evaluation of multiple western blots of protein samples eluted from DNA probes as indicated. The amount of Moi eluted from ssDNA was taken as 1, and the other saturation values are compared to this. Inp: Input; ss: ssDNA; ds: dsDNA; 3′: 3′ overhang; c: no DNA control. Significance level was determined with Student's *t*-test *p < 0.05; **p < 0.01. Two representative Western blots from different gels are shown.

Co-expression of the Ver and Moi proteins in bacteria helped to overcome solubility problems and permitted the study of the DNA binding properties of their dimer. Using biolayer interferometry (figure 1*a*), we found that the Ver–Moi dimer indeed preferably binds ssDNA (figure 1*b*). We detected a weaker interaction with dsDNA as well. The Ver–Moi–ssDNA binding curve reveals binding kinetics that can be described with the heterogeneous ligand model (2 : 1). This suggests that the Ver–Moi complex has two surfaces which bind ssDNA with different kinetics and affinity (figure 1*c*). As the DNA binding property of Ver is known [27] we can assume that the second surface is related to Moi (figure 1*d*).

It would be expected that fast evolution will result in functional anomalies within hybrid complexes formed between proteins of different species. To test the DNA binding of the hybrid dimer formed between *Drosophila yakuba* Ver and *Drosophila melanogaster* Moi we used affinity pull-down experiments. *Drosophila yakuba* and *D. melanogaster* are closely related and able to produce infertile offspring [29]. The amino acid identity between their Ver proteins, however, is only 83.17%, in contrast with some other proteins with conserved functions, such as the centromere-associated protein Borr (96.5%), heterochromatin associated protein HP1a (94,17%), protein kinase Raf1 (99.59%) or cytoskeletal protein Moesin (100%). Nonetheless, the *D. yakuba* Ver–*D. melanogaster* Moi hybrid has similar DNA binding properties as the *D. melanogaster* complex (figure 1*e*).

### 2.2. Structural model of Ver permits identification of important conserved regions

**Figure 2.**
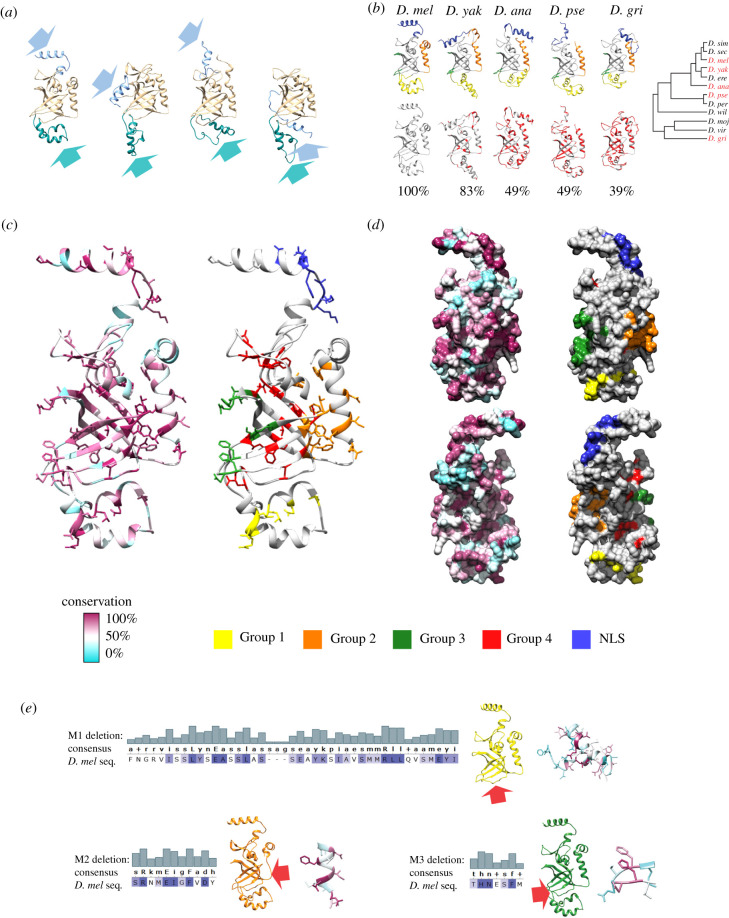
Ver model indicates localization of conserved amino acids in specific interacting surfaces. (*a*) Four models of Ver structure are shown as predicted using Phyre2 [32]. Poorly predicted regions are in light blue and dark cyan, the Ob-fold is in light brown. The N-terminal part (light blue) is attached to the Ob-fold with a loop region. This region was predicted as NLS. The helical structures shown in dark cyan are not part of the Ob-fold barrel and are, therefore, absent from homology-based modelling. The position of the N-terminal helix–loop structure was determined with low confidence, which could be explained by the unstructured nature of the loop. Due to the flexibility of this loop multiple different models were created where the positions of the N-terminal helix in relation to the Ob-fold vary greatly. (*b*) Structural models of Ver protein from different species are shown as predicted by Phyre2. In the first row, similar parts of the models are coloured similarly. In the second row, similarity to *D. mel* sequence is indicated for each model and the differences are coloured red. The phylogenetic tree shows the relation among the studied species. (*c*) Since Ver is considered as an Ob-fold protein we selected a model where the Ob-fold is not affected by low confidence predictions and the N-terminal loop does not bend back to the Ob-fold. Ribbon models and (*d*) surface models are shown. Amino acids are colour coded according to their conservation among Ver proteins of 21 *Drosophila* species. Groups of amino acids can be identified forming conserved surfaces as uncharacterized helices associated surface (Group 1, yellow), Ob-fold supporting helix associated surface (Group 2, orange), putative DNA binding surface (Group 3, green) and Ob-fold (Group 4, red), containing most of the conserved amino acids of Ver. The NLS on the N-terminal of the protein is indicated (NLS, blue). On the surface model on the left conserved regions are shown with maroon–light blue colouring, while on the model on the right these regions are coloured according to their proposed function. The amino acids responsible for the Ob-fold (red) are buried in the structure, therefore, they are not visible on the surface model. (*e*) Mutant versions of Ver were created by deleting the indicated sequences. The conservation values were calculated by using 21 sequences and highlighted with white–blue colour code and histograms. Mutant versions of Ver were also modelled using the first prediction from (*a*) as template with Modeller [33].

The cross-species functional conservation of fast-evolving Terminin proteins suggests that they have conserved interacting surfaces. Sequence level comparison, however, indicates only seemingly sporadic distribution of conserved amino acids, though these might form interacting surfaces when the protein is properly folded. Ver shares similarities with Ob-fold proteins, among which many are able to bind DNA, RNA or proteins as well [30]. Ob-folds, described first in oligosaccharide binding proteins, are domains consisting of a beta barrel of five antiparallel beta strands capped with an alpha-helix [31]. The similarity of Ver to known Ob-fold structures permitted us to create structural models for the protein. This allowed identification of all conserved surfaces along the molecule (figure 2). We were able to predict very similar models using Ver sequences from different species (figure 2*b*).

The Ver structure model indicates that conserved amino acids, which are scattered on the linear sequence, are in positions important for function. Almost all buried amino acids and those known to be responsible for intra-molecular interactions are conserved. The rest of the conserved amino acids are enriched at different surface regions. For the N-terminal region, the structural prediction is poor. This segment is connected to the Ob-fold by a loop structure, suggesting that it is a flexible region (figure 2*a*). This layout is reminiscent of a nuclear localization signal (NLS), which needs to be easily accessible. Indeed, cNLS Mapper software identified this conserved region as an NLS (electronic supplementary material, table S1).

Three further putative interacting surfaces can be identified in the structure. The surface made by amino acids in Group 1 (yellow) is a region with rather poor structure prediction and unknown function. The helix that supports the Ob-fold structure has conserved hydrophobic amino acids that are exposed on the surface (amino acids in Group 2, orange). The last conserved surface is predicted to be responsible for DNA binding, based on its similarities to RPA70 [21]. Amino acids in Group 3 (green) are responsible for forming this region.

### 2.3. The identified surfaces are involved in Ver function

**Figure 3.**
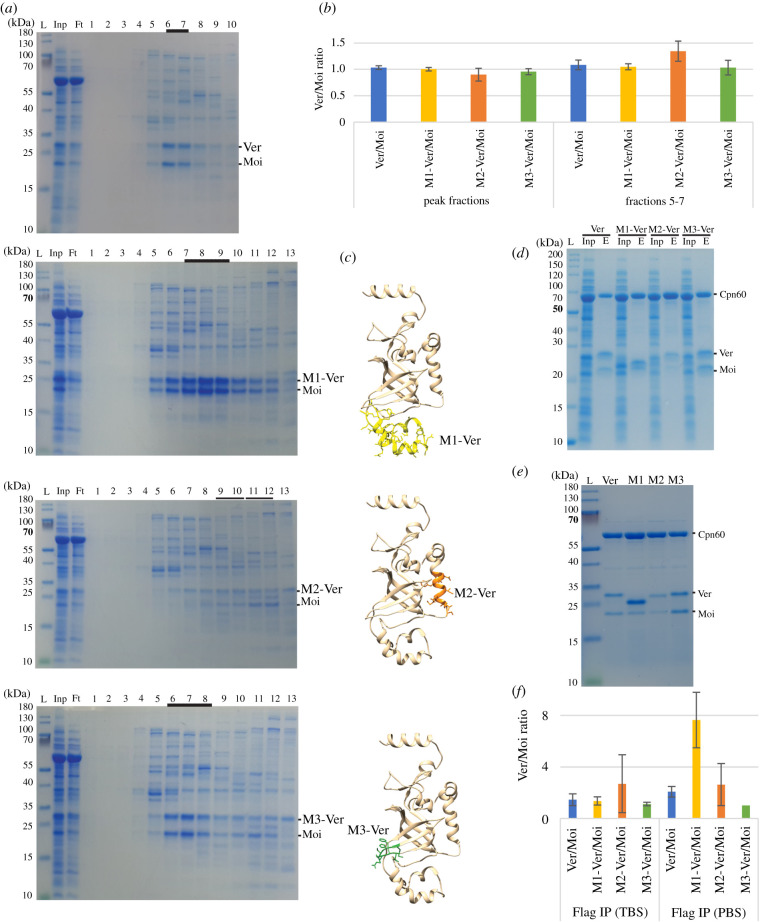
Co-purification of Ver–Moi complexes. (*a*) CCB stained PAGE images obtained by analysing eluted fractions collected during purification of Ver–Moi heterodimers on heparin column. The M1-Ver mutant dimer showed increased binding to the column compared with the wild-type complex. The M2-Ver and Moi eluted at different salt concentrations, suggesting that the deletion affects the Ver–Moi interaction surface. L: molecular weight marker; Inp: input; Ft: flow through; numbers: fractions; underlined: peak fractions. (*b*) Ver : Moi ratio calculated based on pixel intensity is near to one in the peak fractions or in fractions 5–7. M2-Ver : Moi ratio is higher at fractions 5–7 and lower in peak fractions 9–12. (*c*) Models of Ver showing the deleted parts in each mutant. (*d*–*f*) By immune-affinity chromatography both wild-type and mutant Ver proteins co-purified with Moi. However, buffer composition changed the Ver : Moi ratio. In samples that contained M2-Ver less Moi was present. In PBS buffer the proportion of M1-Ver and Moi is also shifted to higher Ver : Moi ratio. Some Cpn60 chaperones also co-purified with the Ver–Moi heterodimers. E: elution.

In order to investigate the role of the identified surfaces of Ver, we generated mutant versions of the protein by introducing deletions into a Ver–Moi bicistronic pET vector. We completely removed the uncharacterized helixes (Group 1—M1-Ver) or partially deleted the Ob-fold supporting helix (Group 2—M2-Ver) and the DNA binding surface (Group 3—M3-Ver; figures 2*e* and 3*c*), while leaving the Moi coding sequence unchanged. Ver and its mutant versions were N-terminally tagged with Flag, and Moi was tagged at its C-terminal with haemagglutinin (HA), in order to facilitate immunodetection of the proteins on western blots. Additionally, the Flag tag on the N-terminal part of Ver also serves as an epitope for immune-affinity purification. The proteins were co-expressed in bacteria and co-purification of the subunits, as an indicator of complex assembly, was studied (figure 3*b*).

During heparin-sepharose column chromatography of cell lysates containing wild-type Ver and Moi, dimers of the proteins eluted at low salt concentration at 280–325 mM (in fractions 6 and 7). Deletion of the Group 1 surface of Ver (M1) did not affect its interaction with Moi as the proteins eluted together. However, the affinity of M1-Ver–Moi dimer to the column increased due to the deletion, as the dimer eluted at a higher salt concentration (between 325 and 415 mM in fractions 7–9) than the wild-type proteins.

Deletion of the Group 2 region resulted in a lower yield of soluble proteins; furthermore, it weakened Ver–Moi interaction as M2-Ver co-purified with Moi only partially and the majority of the two proteins eluted at different salt concentrations. The elution pattern of M3-Ver mutant protein (Group 3) was similar to that of M2-Ver; however, the stability of the dimer was affected less, since the majority of the proteins co-eluted at 280–325 mM (fractions 6 and 7).

During Flag immune-affinity purification wild-type Ver, M1-Ver and M3-Ver mutant proteins co-purified with Moi near 1 : 1 stoichiometry. M2-Ver mutant on the other hand bound less Moi (figure 3*d*,*f*). A significant amount of Cpn60 chaperone also co-purified with these proteins. Curiously a buffer change from TBS to PBS altered the Ver/Moi ratio of the complexes (figure 3*e*,*f*).

In order to determine whether the deletions influenced the stability of Ver, we performed thermal unfolding experiments using immune-purified protein samples (see electronic supplementary material, figure S2). Ver and Moi both contain one tryptophan residue, therefore their transition from hydrophobic to hydrophilic environment could be tracked based on fluorescence intensity changes at 350 and 330 nm. (The Cpn60 present in the protein samples contains no Trp residue, therefore it does not produce significant signal in these assays.) In wild-type heterodimers, we detected two transition events corresponding to the denaturation of Ver or Moi. The M1-Ver mutant showed identical and the M3-Ver mutant a similar denaturation event to the wild-type Ver. In the case of the M2-Ver mutant, the first event was not detected since the deletion altered the position of the Trp residue that provided the signal in other versions of Ver.

**Figure 4.**
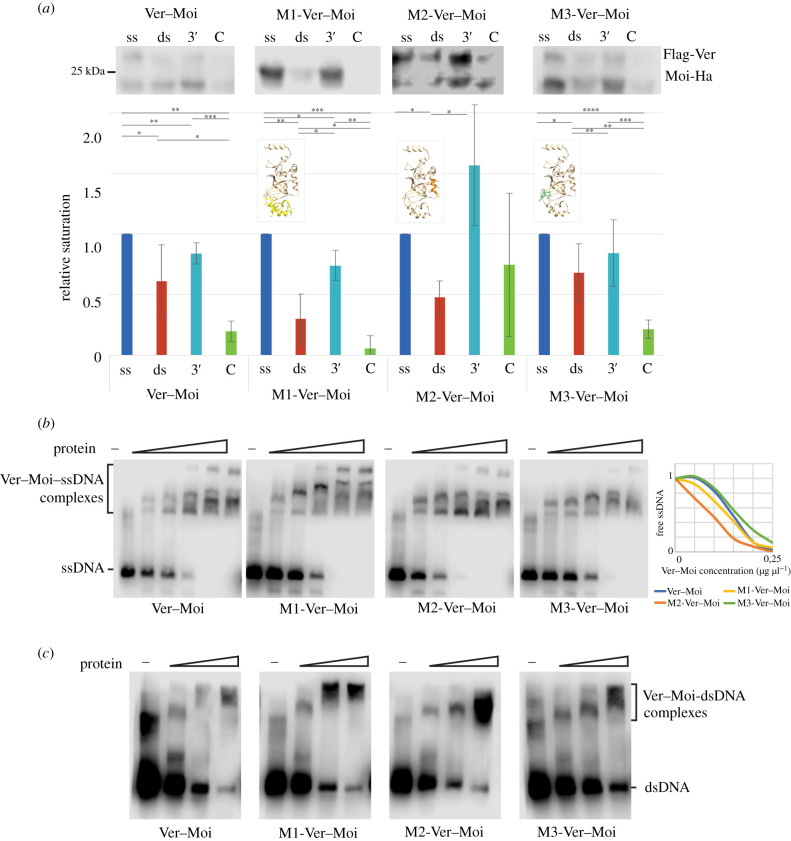
DNA binding affinity of mutant Ver–Moi complexes. (*a*) Western blots of protein samples eluted in DNA affinity pull-down experiments. Both Ver and Moi western blots are shown, and the graph shows qualitative evaluation of the results. Each studied mutant binds preferentially to ssDNA. Student's *t*-test was applied to determine significance levels. *p < 0.05; **p < 0.01; ***p < 0.001; ****p < 0.0001. Four representative western blots from different gels are shown. (*b*) Electrophoretic mobility shift assays of Ver mutants and ssDNA probe. Each Ver mutant binds ssDNA with similar affinity and Ver–Moi–ssDNA complexes with different mobilities are formed. In the binding assays shown a five step 1 : 2 dilution series of a 0.25 µg µl^−1^ Ver–Moi protein samples was used. The graph on the right shows changes in free ssDNA determined as the average of three independent EMSA experiments. (*c*) EMSA of Ver–Moi complexes with dsDNA probe. Wild-type and mutant Ver containing complexes bind dsDNA with similar affinity. In the assay shown three steps 1 : 3 serial dilution of 0.25 µg µl^−1^ Ver–Moi protein samples was used.

To determine the effect of Ver mutations on the DNA binding of Ver–Moi dimers we performed DNA affinity pull-down experiments (figure 4*a*). We found that the mutations did not cause major changes in DNA preference and most heterodimers bound to ssDNA. Comparisons between binding affinities of mutants by this technique was, however, complicated to some extent by the fact that low levels of binding were also detected on control empty beads. Therefore, we used another technique to evaluate the binding affinity of mutants. Results of electrophoretic mobility shift assays (EMSA) did not indicate significant differences in the binding affinities of wild-type and mutant Ver–Moi dimers to ssDNA (figure 4*b*). The mutations did not abolish DNA binding and each mutant formed DNA–protein complexes with various mobility in a concentration-dependent manner. Our trials to identify protein components of the complexes by detecting supershifts with the use of antibodies for Ver and Moi unfortunately did not produce clear results. Nonetheless, the multiplicity on detected complexes supports the assumption of multiple DNA binding surfaces within the heterodimer.

## 3. Discussion

Subunits of Terminin are among the fastest evolving proteins of *Drosophila* [20,34]. Recently, a molecular cause that might explain this has been suggested in the utilization of telomeric retrotransposons, which are required for telomere maintenance [35]. Since transposons evolve fast due to the low fidelity of their reverse transcriptase, the proteins that keep their replication at bay must also have fast evolution. A similar phenomenon is seen in the case of viruses and proteins involved in the response to viral infection [35,36]. The positive selection could eventually result in divergent evolution, which decreases the compatibility of proteins from different species [28]. Similarly, the coevolution of telomeric proteins and their DNA targets might also have interesting implications regarding species evolution [25,37].

**Figure 5.**
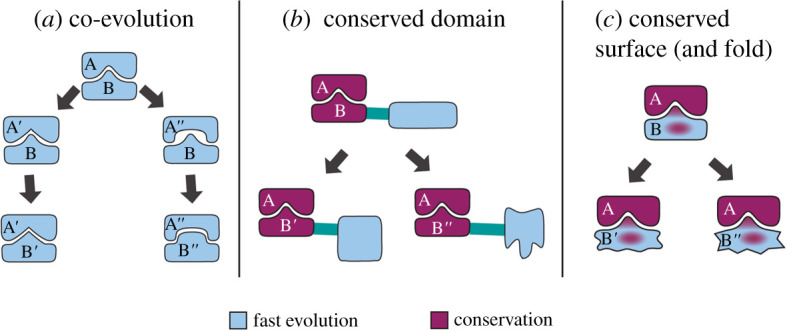
Different scenarios to preserve interactions between fast-evolving proteins. (*a*) Two independent mutations in protein A result in A′ and A″ variants with altered interacting surfaces with protein B. New mutations and positive selection result in B′ and B″, preserving specific interaction with A′ and A″, respectively. As a result of coevolution, interactions between ′ and ″ proteins are less and less likely. (*b*) Fast-evolving protein A and B might preserve interaction via conserved domain(s), which do not change rapidly in contrast with the rest of the proteins. (*c*) Despite their fast evolution in overall sequence, the amino acids that allow the correct fold and play key roles in interacting surface(s) are preserved in protein A and its partner B.

It is puzzling how *Drosophila* telomere-associated proteins are able to maintain their function despite massive changes in amino acid composition [18,25]. One can envision two general scenarios by which interacting proteins can preserve affinity towards each other (figure 5). The first could be that coevolution maintains the conserved function since a change in one molecule is paired with a compensatory change in its interacting partner. Indeed, fast-evolving proteins can co-evolve and mutual changes in the interacting partners keep their complexes intact [38,39]. Assuming this scenario, the preservation of interaction between fast-evolving homologues from different species is not expected. However, we could show interaction between *D. yakuba* Ver and *D. melanogaster* Moi proteins. The existence of functional hybrid complexes such as the *D. yak*-Ver–*D. mel*-Moi suggests that coevolution is not the only way to preserve interaction between fast-evolving molecules. Another possible mechanism could be that interacting surfaces needed for function are not affected by the accelerated evolution. The speed of evolution of different protein domains even within the same protein can differ greatly [25]. Disordered regions like hinges and loops usually change much faster than globular parts [40]. Thus, a fast-evolving protein might consist of extensive disordered regions and conserved region(s) which preserve function. In theory, conserved folds and surfaces are sufficient to maintain interaction between proteins. Fast evolution at protein regions forming loop structures is commonly detected [40], since multiple amino acids could be substituted in loops without destroying the structure. Similarly, multiple alpha-helix- or beta-sheet-forming amino acids could be interchanged without causing damage to the secondary structure. Consequently, as long as the structure is not affected by the mutations, the interacting surfaces are also preserved (figure 5).

The structural model of Ver shows conserved hydrophobic amino acids buried inside the structure (figure 2*c*,*d*). This hydrophobic core most probably facilitates the correct positioning of the beta-sheet elements contributing thus to the Ob-fold formation. Modelling of Ver structures from different species predicts similar folds, indicating the conservation of the Ob-fold despite significant differences in amino acid sequences (figure 2*b*). Other conserved amino acids positioned on the surface can be involved in intermolecular interactions. In the structural model of Ver, in addition to a conserved surface on the N-terminal that we propose to be a NLS, we identified three regions that deserve attention as possible determinants of interactions with DNA and/or Moi. Ver was described to bind DNA as a homodimer [27], but was also suggested to be functional only as a member of the MTV complex [26]. We found that Ver–Moi heterodimer is able to bind ssDNA in the absence of Tea. Thus, our result supports a role of the Ver–Moi complex in telomere organization in association with ssDNA and questions the role of Tea in DNA binding. Tea might have a chaperone-like function, in which it was replaced by Cpn60 in our experimental setup. According to our data, the Ver–Moi dimer has two DNA binding surfaces, suggesting that Moi also has DNA binding activity. The low solubility of Moi, however, precludes direct detection of its DNA binding by affinity pull-down. Nevertheless, apart from the results of BLI and EMSA, the results of Far western experiments also indicate that Moi is able to bind DNA. In our experiments Moi as well as Ver–Moi dimer interacted with H1 histone. However, Moi interaction with H1 was DNA dependent. Overall, these results support that Moi also has affinity to bind DNA.

A deletion we introduced in the putative DNA binding surface of Ver had minimal effect on the DNA binding properties of the Moi–Ver complex *in vitro.* This could indicate that the DNA binding surface is much larger than we anticipated, or another surface is responsible for the interaction with the DNA. The crystal structure of the CDC13 protein indicates the involvement of a large surface in DNA binding as the ssDNA wraps around half of the Ob-fold domain [41]*.* The stability of the M3-Ver mutant was slightly different from that of wild-type Ver, suggesting that the mutation caused minor alteration in the fold of the protein. This could also explain the slight difference observed in the elution profile from the heparin-sepharose column.

Deletion of the uncharacterized helices (Group 1) (M1-Ver) resulted in a Ver with higher affinity to heparin but the similar affinity to DNA (figures 3 and 4). Though the deletion increased the solubility of the protein, the thermo stability of M1-Ver and wild-type Ver was identical, indicating that the Ob-fold was not affected by this mutation.

Deletion of the Group 2 region (M2-Ver) caused the most serious alterations in Ver molecular behaviour. In affinity pull-down experiments, a stronger binding to beads resulted in a change in elution profile; however, the complex was still able to bind DNA. For the C-terminal helix, a role in Ver–Ver dimerization has been suggested, and it was shown that a mutation in it prevents Ver from forming homodimers and as a result, binding DNA [27]. The proposed surface of Ver–Ver interaction is not the conserved surface in our model. Flag immuno purification indicated that M2-Ver interacted with Moi, during heparin-sepharose column chromatography, however, M2-Ver and Moi did not co-purify. This indicates that the C-terminal helix has an important role both in homo- and heterodimer formation. Since Ver preferentially forms heterodimers in the presence of Moi [25,26] and a deletion in the C-terminal helix diminished both Ver–Ver [27] and Ver–Moi interactions, we conclude that surfaces involved in these interactions extend to the C-terminal helix and at least partially overlap.

In summary, we showed that the structure predictions of rapidly evolving Ver protein from different species resulted in similar folding despite large differences in the sequences (figure 2*b*). The hydrophobic amino acids buried in the folded protein structure are conserved (figure 2*c*,*d*), suggesting that a limited conservation of specific amino acids is sufficient to maintain the correct shape of the protein. Some of the selected surfaces can be well associated with functions (N-terminal: NLS; Group 2: Moi-interacting surface). The role of further preserved surfaces might be in interactions with other Terminin and related proteins (Tea, Nap1, SSe, RPA70 or CG7341 [21,26,27,42]).

**Figure 6.**
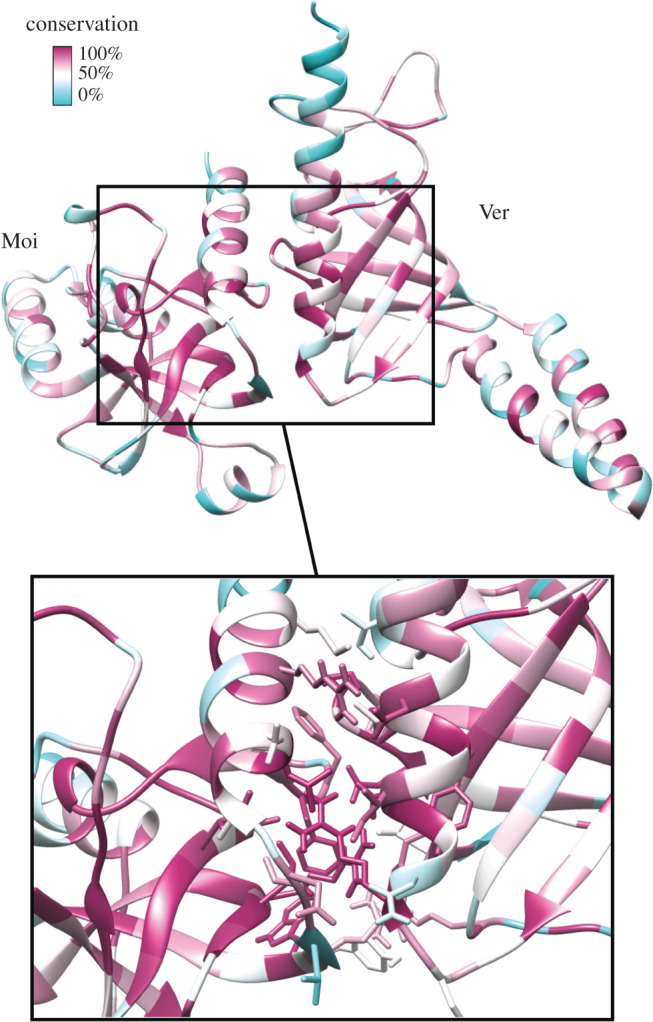
Ver–Moi dimer docked *in silico.* In the model the interacting surfaces consist of conserved amino acids.

Due to the highly accurate protein structure predictions available in the AlphaFold database [43] we could conclude that similarly to Ver, Moi is also an Ob-fold protein (electronic supplementary material, figure S3), and we were able to dock Moi to Ver by taking advantage of its high resemblance to the Stn1–Ten1 dimer (electronic supplementary material, figure S3). The model fits with our experimental data since M2 mutation partially affects this surface. Most of the amino acids that participate in the interaction are conserved (figure 6). Thus, *in silico* structure predictions and the existence of a functional hybrid complex support our hypothesis that the preservation of Ver–Moi function in the fast-evolving Terminin complex is the result of the conservation of amino acids at key positions. These amino acids ensure the correct protein folding and by that allow the formation of the proper interacting surfaces.

## 4. Methods

### 4.1. Cloning and heterologous protein expression

To generate mutant versions of the Ver protein, alterations were introduced into the coding region by PCR mutagenesis in previously constructed expression constructs [25]. Primers used for this are listed in electronic supplementary material, table S2.

Protein production was done in Arctic express cells, DE3 (Agilent Technologies) at 10–18°C, with induction by 0.3 mM IPTG final concentration for 24–60 h.

Moi-HA cDNA was cloned in pAcUW21 vector using classic molecular biological methods. HA-Moi expressing baculovirus strain was constructed using the Baculovirus Gold kit (BD Biosciences). The viral clones were selected by the dilution method. The most promising strains were used to infect Sf9 cells. The cells were grown in TNM-FH insect culture medium (Sigma), supplemented with 10% FCS and antibiotics and collected 3 days after infection.

### 4.2. Protein purification

Bacterial cells were lysed by sonication using Sonics Vibra cell apparatus. Each sample was sonicated by six cycles, 20 s each with 10 s breaks at 45% amplitude in buffer I (25 mM Tris HCl pH 7.5, 100 mM NaCl, 1 mM CaCl_2_, 1 mM MgCl_2_).

Cell extracts were cleared by centrifugation and filtration through G75 Sephadex beads (GE Healthcare) and loaded to the heparin-sepharose column (GE Healthcare). Proteins were eluted by a 0.1–1 M NaCl gradient in 20 mM Tris pH 7.5 in 20 ml and 1 ml fractions were collected.

Relevant fractions from the heparin-sepharose column were loaded to the anti-FLAG M2 affinity matrix (Sigma-Aldrich) to perform immune-affinity purification. The beads were washed with buffer I or PBS (137 mM NaCl, 2.7 mM KCl, 10 mM Na_2_HPO_4_, 1.8 mM KH_2_PO_4_). Elution was performed with 150 µg ml^−1^ 3× Flag peptide (Sigma) in buffer I or PBS.

### 4.3. Gel electrophoresis and Western blot

Proteins were separated on 10% tricine–SDS–PAGE [gel buffer (1 M Tris, 0.33 M HCl, 0.1% SDS pH 8.45), anode buffer (0.1 M Tris, 0.022 M HCl, pH 8.9), cathode buffer (0.1 M Tris, 0.1 M Tricine, 0.1% SDS, pH 8.25)] [44]. For visualization gels were stained in Coomassie brilliant blue or western blot was performed.

For western blot, the proteins were transferred to a nitrocellulose membrane (0.45 um, GE Healthcare). For blocking the membrane low-fat milk (5%), as primary and secondary antibodies Rabbit anti-HA (Abcam, Ab9110) or mouse anti-Flag M2 (Sigma F3165) and HRP conjugated antibodies (Dako P0448, P0260) were used, respectively. Immobilon Chemiluminescent HRP substrate (Millipore) and Li-Cor western Blot scanner were used to detect light signals. Western blots were quantitatively evaluated by ImageJ. The uncropped western blots are shown in electronic supplementary material, figure S3.

### 4.4. Determination of thermal stability

The thermal stability of the Ver–Moi complexes were determined with Prometheus NT.48 (Nanotemper) in buffer I.

### 4.5. DNA binding

DNA binding assays were based on immobilization taking advantage of the fact that biotin labelled oligonucleotides bind effectively to streptavidin-coated surfaces. An 80 nucleotide (nt) long biotin labelled single-stranded DNA with low propensity to form secondary structures was generated and with the use of an oligonucleotide with complementary sequence (electronic supplementary material, table S2), double-stranded (ds) DNA (80 nt) or dsDNA with a 3′ overhang structure (40–40 nt) bound to the same matrix was formed.

For affinity pull-down assay biotinylated DNA was bound to streptavidin covered magnetic beads (Dynabeads M280 Streptavidin, Thermo). Partially purified Ver–Moi dimers eluted from the heparin-sepharose column were incubated with the oligonucleotide-containing beads at 4°C, overnight. The beads were washed with buffer I, and the bound proteins were eluted with a buffer that contained 1 M NaCl, 25 mM Tris HCl pH 7.5, 1 mM CaCl_2_, 1 mM MgCl_2_, and analysed by western blot. The blots were evaluated with ImageJ. Two-tailed paired Student's *t*-test was applied to calculate significance.

For Biolayer interferometry biotinylated oligonucleotides (3 µg ml^−1^) were bound to a streptavidin covered sensor (SAX sensor, Fortébio). Protein samples were prepared in PBS buffer (137 mM NaCl, 2.7 mM KCl, 10 mM Na_2_HPO_4_, 1.8 mM KH_2_PO_4_) supplemented with 0.05% Tween 20. After the binding step the surface was blocked with 10 µg ml^−1^ biocitin (Sigma), the baseline was measured in the buffer and the data was collected. We used the Octet system (FortéBio) and its software for evaluation.

EMSA were performed using LightShift Chemiluminescent EMSA Kit (ThermoFisher). In total, 20 fmol biotinylated ssDNA was used in each reaction. Ver–Moi protein concentrations varied from 5.55 to 0.35 µM, produced by two-fold serial dilution. Binding reactions were done in PBS buffer (137 mM NaCl, 2.7 mM KCl, 10 mM Na_2_HPO_4_, 1.8 mM KH_2_PO_4_) supplemented with 0.05% Tween 20, for 15 min at room temperature. Electrophoresis was performed on 7.5% acrylamide gel in 0.5× TBE buffer. Signals were detected following the instructions of the manufacturer using Li-Cor western blot scanner.

### 4.6. Sequence analysis and three-dimensional modelling

Ver three-dimensional structures were built using Phyre2 [32]. The model was visualized by UCSF Chimera [45]. The conservation of the amino acids was calculated based on 21 *Drosophila* orthologues [25]. The NLS was determined by the cNLS Mapper software using 3.0 cut-off value [46]. Structures predicted by the machine-learning algorithm were obtained from AlphaFold Protein Structure Database [43]. Protein docking was performed on the HADDOCK2.4 webserver using basic settings [47,48].

### 4.7. Far western

Query proteins were separated on tricine–SDS–PAGE, transferred to a nitrocellulose membrane and the membrane was soaked in buffer (5% milk powder, 20 mM Tris pH 7.5, 100 mM NaCl, 1 mM MgCl_2_, 1 mM CaCl_2_) to prevent nonspecific binding of the probe. The HA-Moi protein probes were produced in Sf9 cells using recombinant baculovirus. Ver–Moi-HA heterodimer probes were expressed in bacteria. Cell extracts of both Sf9 and bacterial cells were prepared by sonication, cleared by centrifugation and used as probe. The extracts were mixed at 1 : 9 ratio with binding buffer (with or without Benzonase (Merck)) and incubated on the membrane overnight at 4°C. Next, the membrane was washed in TBST (20 mM Tris, 150 mM NaCl, 0.05% Tween 20) and incubated with the primary antibody specific for the HA epitope present on the probe (Abcam, Ab9110). Further steps were done as those described for western blots.

## Data accessibility

This article has no additional data.
